# Changes in self-esteem, body dissatisfaction, and body appreciation during face-to-face and digital supervised physical activity interventions in women with obesity: A dual-study investigation

**DOI:** 10.1016/j.obpill.2026.100303

**Published:** 2026-07-16

**Authors:** Lisa Moyon, David Le Foll, Alexandre Mazéas, Geneviève Cabagno

**Affiliations:** a University of Rennes, UR2, Laboratoire Valeurs, Innovations, Politiques, Socialisations et Sports (VIPS^2^) - UR 4636, Place du recteur Henri Le Moal, Rennes, 35000, France; b University of the West - Institute for Training in Physical Education and Sport of Angers (UCO-IFEPSA), APCoSS, 49 Rue des Perrins, Les Ponts-de-Cé, 49130, France; c University of Southern Denmark, Department of Sports Science and Clinical Biomechanics, Campusvej 55, Odense, DK-5230, Denmark; d University of Southern Denmark, Danish Centre for Motivation and Behaviour Science (DRIVEN), Odense, DK-5230, Denmark

**Keywords:** Body appreciation, Body dissatisfaction, Digital intervention, Exercise, Physical self-perceptions, Physical subdomains

## Abstract

**Background:**

Physical activity (PA) is a cornerstone of obesity treatment. However, changes in self-perceptions during supervised PA interventions remain insufficiently understood, particularly regarding their temporal dynamics and maintenance after intervention completion. Moreover, because individuals with obesity tend to avoid in-person PA due to fear of stigma, digital PA programmes may represent a particularly relevant alternative.

**Methods:**

This article reports a multi-time-point intervention study using a dual-independent cohort design. Changes in self-esteem and body image facets during supervised PA interventions in women with obesity were explored in two independent longitudinal studies. Global self-esteem (GSE), physical self-worth (PSW) and its four subdomains (sport competence, physical condition, physical strength and body attractiveness), body dissatisfaction and body appreciation were assessed at baseline, monthly during each intervention, and at three-month follow-up.

**Results:**

In Study 1, women who completed the face-to-face intervention (n = 63) showed significant increases in GSE, PSW and its four subdomains, and body appreciation, along with a significant decrease in body dissatisfaction. In Study 2, women who completed the digital intervention (n = 19) showed similar patterns of change, except that GSE, physical strength, and sport competence did not significantly differ from pre-to post-intervention. Across both studies, most changes were observed within the first month, plateaued over the remaining two months, and were maintained at follow-up. A similar pattern was observed across both modalities.

**Conclusion:**

These findings suggest that supervised PA interventions, delivered either face-to-face or digitally, may be associated with improvements in self-esteem and body image among women with obesity and that the first weeks of a supervised PA programme may represent a critical moment for these variables. These findings are relevant in the context of the obesity epidemic, as these variables are key indicators of mental health and predictors of healthy behaviours and long-term weight loss.

## Graphical abstract

**Figure:**
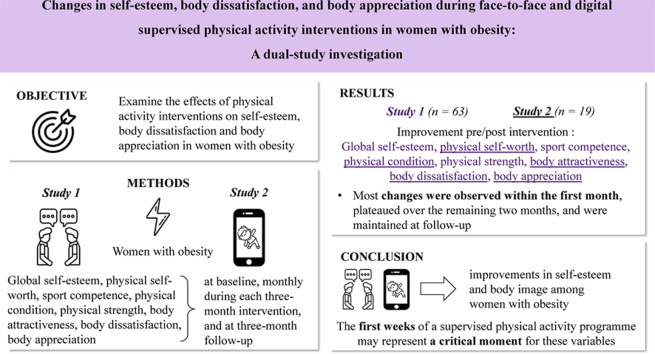

## 1 Introduction

Obesity has emerged as a major public health issue due to the epidemic rise in its prevalence worldwide. While the number of individuals with this condition has tripled since 1975 [1], it is estimated that one in seven men and one in five women will be affected by 2030 [2]. Women living with obesity are particularly vulnerable to weight discrimination and face a heightened risk of impaired mental health [3].

As an indicator of mental health, self-esteem is a hierarchical, multidimensional construct that reflects an individual's subjective evaluation of their worth as a person [4]. At the apex is positioned global self-esteem (GSE), which is shaped by several domains, including physical self-worth (PSW). PSW comprises four subdomains reflecting self-perceptions in terms of sport competence (SPORT), physical condition (COND), physical strength (STREN) and body attractiveness (BODY). SPORT assesses perceived sport skill and learning, COND reflects perceived fitness and endurance, STREN assesses perceived muscular strength and development, and BODY evaluates the perceived attractiveness of the body and the ability to maintain it. Interactions between the apex, the domains, and the four subdomains occur in both bottom-up and top-down directions [5].

Body image also plays an important role in mental health. This multidimensional concept encompasses two key facets: body dissatisfaction, defined as the degree of dissatisfaction an individual feels about their physical appearance [6] and body appreciation, a favourable, accepting stance toward the body's features, functions, and health [7]. Each of these facets is influenced by interpersonal relationships and experiences [[8], [9], [10]].

These constructs are particularly relevant to obesity management, as higher body mass index (BMI) is typically linked to lower GSE, PSW (and its subdomains), body appreciation, and to higher body dissatisfaction [[11], [12], [13], [14], [15], [16]]. Thus, these relations may influence long-term weight loss and, consequently, obesity management outcomes. Conversely, research has shown that higher self-esteem and a functional body image help individuals reduce the risk of mental disorders (e.g., eating disorders [17]) and adopt healthy behaviours (e.g., engagement in physical activity [18]), promoting well-being and better health. Supporting individuals with obesity in improving their self-esteem and body image is therefore a priority of obesity management.

Such support could be delivered through physical activity (PA) programmes, which, when combined with other behavioural strategies, stand out as one of the cornerstones of obesity treatment. Beyond its effect on BMI reduction and body composition [19], PA is known to benefit mental health [20], making it a promising tool to enhance self-esteem and body image. For instance, Cramer et al. [21] identified the benefits of practicing yoga in the form of a 12-week face-to-face supervised PA programme on GSE in women with obesity. However, the effects on PSW and its four subdomains were not investigated, which inherently limits our understanding of the underlying mechanisms at play. To our knowledge, the study conducted by Megakli et al. [12] is the only one that has explored the question in this population, specifically considering PSW and its four subdomains rather than just GSE. The results of this longitudinal study showed that a 12-week face-to-face PA programme could significantly increase GSE, PSW, and its subdomains – except for BODY. The authors also reported monthly non-linear changes throughout the programme. Nevertheless, the temporal evolution of these psychological variables during supervised PA interventions in women with obesity has received limited attention and warrants further investigation.

In addition, the impact of face-to-face supervised PA programmes on body image – particularly on body appreciation in women with obesity – remains largely unexplored. While Carraça et al. [22] demonstrated that a multimodal cognitive-behavioural intervention including PA could reduce body dissatisfaction at 12 and 24 months, it remains unclear whether similar effects could be identified earlier in the intervention process. Moreover, their study did not include body appreciation as an outcome. Carraça et al. [23] more recently emphasized that the effects of a supervised PA intervention on self-perceptions in individuals with obesity remain poorly, if not entirely, unstudied – highlighting a critical need for further research in this area. Moreover, a month-by-month analysis must be implemented, along with a follow-up phase to collect post-intervention data, which is currently lacking in the literature to understand its dynamics [23]. This data, collected from participants attending the intervention in person, could help offer more tailored and appropriate support to this population. However, it also highlights the need to explore alternative approaches for individuals who face barriers to in-person attendance, thereby ensuring that all patients can benefit from optimized support.

Beyond face-to-face programmes, these questions are also relevant in the context of remote and hybrid PA programmes, which have gained popularity in recent years [24,25]. On the one hand, the mechanisms through which face-to-face PA programmes may improve self-esteem and body image, such as direct supervision, embodied interaction, and in-person social support, may not automatically transfer to digital formats, particularly when remote interventions provide limited human support or fail to sustain engagement [26,27]. On the other hand, digital interventions could facilitate the participation of stigmatized individuals, who are often subject to stereotypes (e.g., those with obesity), by offering a relatively more anonymous environment than in-person settings [28], while still enabling interaction with peers online (e.g., discussion forums [29]). Because many people with obesity avoid in-person PA environments due to fear of stigma, digital PA programmes may be particularly beneficial for this population [30]. These interventions have already demonstrated their effectiveness in helping individuals with obesity adopt a healthy lifestyle and lose weight [31]. Digital formats may therefore confer benefits by reducing the fear of stigmatization while fostering interaction and overcoming logistical barriers, ultimately enhancing self-efficacy related to PA, promoting physical changes, and contributing to improvements in self-esteem and body image. However, to our knowledge, changes in self-esteem and body image have not yet been explored in the context of digital PA interventions.

### 1.1 Purpose of study 1 and study 2 and hypotheses

In summary, although supervised PA interventions appear promising for supporting self-esteem and body image in women with obesity, the existing literature remains limited in several important ways. First, few studies have examined changes in both global and physical self-perceptions during PA interventions, and even fewer have considered their temporal dynamics through repeated measurements across the intervention period and follow-up. Second, the effects of PA interventions on body image, particularly body appreciation, remain insufficiently documented in this population. Third, while digital PA interventions may help overcome barriers associated with in-person participation, their potential effects on self-esteem, body dissatisfaction and body appreciation have not yet been examined. To address these gaps, we conducted a multi-time-point intervention study using a dual-independent cohort design with two independent longitudinal studies, each focusing on a three-month supervised PA intervention for women with obesity. Study 1 examined a face-to-face PA programme, whereas Study 2 explored a digital PA programme to understand whether and how each intervention was associated with changes in self-esteem and body image over time.

The first main objective was to examine, across two separate studies, the effects of a three-month face-to-face PA intervention and a three-month digital PA intervention on GSE, PSW and its four subdomains, body dissatisfaction, and body appreciation in women with obesity, while analysing the impact of BMI and age on these changes. As all participants within each study were enrolled in a single intervention group, the aim was not to compare intervention modalities or to infer causal effects, but rather to describe changes within each study separately. While the two studies share identical outcome measures, duration, and analytical strategy, they were designed as independent investigations rather than a formal comparative trial. The differences in delivery modality were accompanied by inherent differences in session frequency, equipment access, and social dynamics that preclude direct statistical comparison. Nevertheless, we propose to examine and discuss both studies within a single manuscript for a richer investigation of how supervised face-to-face and digital PA programmes may influence self-perceptions in this population. For Study 1, we hypothesized that, compared with baseline, participants would report higher levels of GSE, PSW, COND, SPORT, STREN, and body appreciation at the end of the programme, as well as lower levels of body dissatisfaction. Based on previous findings [12], no significant change was expected for BODY. Given its exploratory nature, Study 2 did not test predefined hypotheses but aimed to generate preliminary evidence regarding changes in these psychological outcomes during a digital PA intervention. A secondary objective in both studies was to examine the month-by-month trajectories of change in each psychological variable throughout the programme, as these changes may follow non-linear patterns.

The second main objective was to determine whether any changes observed during the intervention were maintained three months after its completion, again while accounting for BMI and age. For Study 1, we hypothesized that GSE, PSW, and BODY would remain stable at follow-up, whereas COND, SPORT, and STREN would decrease, as these dimensions may be more directly related to ongoing PA practice. For Study 2, given its exploratory nature, no predefined hypotheses were formulated; instead, the study aimed to provide preliminary insights into the evolution of these psychological outcomes over time.

## 2 Study 1

### 2.1 Study 1 methods

Study 1 was a prospective longitudinal observational cohort study evaluating changes during participation in an existing face-to-face supervised physical activity programme.

#### 2.1.1 Participants and procedure

**Fig. 1:**
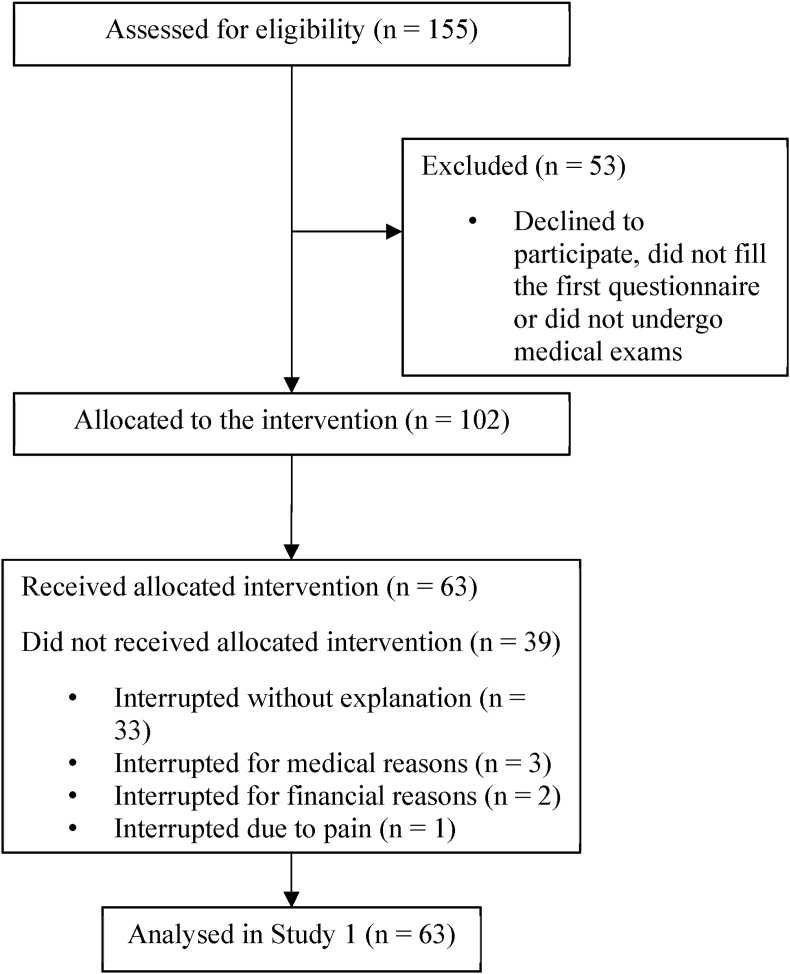
Participants flowchart for the face-to-face intervention.

Inclusion criteria were as follows: 1) being a woman, (2), being at least 18 years, (3) being treated for obesity (i.e., BMI ≥30 kg/m^2^), and (4) being committed to the SporMed supervised PA programme for three months with a medical prescription. An additional inclusion criterion was sufficient proficiency in French. Non-inclusion criteria were pregnancy and physical inability. Participants who did not complete the entire three-month programme (dropout rate = 38.2 %; see Fig. 1), defined as no participation in sessions during the last month, were not included in the final analyses.

For ethical reasons, a waitlist or no-intervention control group was not included, as it was considered inappropriate to ask individuals seeking treatment for obesity to temporarily forgo a clinically prescribed, beneficial intervention. This decision is consistent with precedent in similar intervention research (e.g. Ref. [32]). To partially mitigate the absence of a control condition, we employed a longitudinal repeated-measures design with five assessment points, allowing us to model within-person change trajectories rather than relying solely on pre-post comparisons.

Before entering the supervised PA programme at the SporMed Sports Medicine and Exercise Rehabilitation Centre (Rennes, France), eligible participants were contacted by the first author and informed about the study objectives, procedures, potential risks and benefits, as well as their right to withdraw from the study at any time without consequences. Electronic informed consent was obtained from all participants who fulfilled the inclusion criteria prior to data collection.

To ensure transparency, the TIDieR reporting guidelines were followed. This study was approved by Rennes 2 ethics Committee (n°2019-019), and a preregistration was conducted (available here: https://aspredicted.org/N5D_KG2). Following a longitudinal design, the dependent variables were measured using an online LimeSurvey® questionnaire administered to each patient entering the programme on five occasions: at baseline before initiation of the exercise programme (T0), after each month of the programme (T1, T2 and T3), and three months after the end of the programme, i.e., as a follow-up (T4). Data were collected between October 2022 and December 2023.

#### 2.1.2 Measures

*Demographic, anthropometric variables*. Information on age, weight, and height were collected at T0, both self-reported and via bioelectrical impedance analysis, allowing for the calculation of BMI as follows: BMI (kg/m^2^) = weight (kg)/height (m)^2^. A comparative analysis between BMI calculated from self-reported and measured data was performed, which confirmed a low risk of underestimation (p = .353). Self-reported weight was then collected at each measurement time to calculate BMI.

*Global self-esteem and physical self-perceptions*. A French version (PSI: Physical Self-Inventory [33]) of the Physical Self-Perception Profile (PSPP [4]) was used for this study. This original short form questionnaire consisted of 12 items assessing 6 areas: global self-esteem (GSE), physical self-worth (PSW), physical condition (COND), sports competence (SPORT), physical strength (STREN), and attractive body (BODY). Each component was measured using two items. This questionnaire was based on a 6-point Likert-type response scale with response options ranging from 1 (not at all) to 6 (entirely). Higher scores reflected a greater perception of GSE, PSW and its subdomains. Internal consistencies (Cronbach's alpha) for the present study were acceptable (0.67, 0.84, 0.66, 0.81, 0.76, and 0.85 for the GSE, PSW, COND, SPORT, STREN, and BODY components respectively), confirming the reliability of the French version of the questionnaire.

*Body dissatisfaction*. Body dissatisfaction was assessed using the French version [34] of the Body Shape Questionnaire-8C (BSQ-8C; Welch et al., 2012) developed by Cooper et al. [35]. This questionnaire consists of 8 items that capture respondents’ concerns about the shape and size of their body over the past 4 weeks. Participants were asked to respond using a 6-point Likert scale ranging from 1 (never) to 6 (always). A higher score indicates a greater level of body dissatisfaction. For this study, the internal consistency was good (α = 0.84), confirming the reliability of the French version of the questionnaire.

*Body appreciation*. Body appreciation was measured using the French version [36] of the revised 10-item Body Appreciation Scale-2 (BAS-2 [37]) based on the original work of Avalos et al. [38]. The items, which assess acceptance and/or positive attitudes towards the body, were rated on a 5-point Likert scale ranging from 1 (never) to 5 (always). A higher score indicates a greater level of body appreciation. For this study, the internal consistency was excellent (α = 0.93), confirming the reliability of the French version of the questionnaire.

*Adherence*. Participants were asked to report the number of sessions they attended over the past month at T1, T2, and T3.

#### 2.1.3 Description of the PA intervention

Participants benefited from an existing 12-week supervised PA programme (i.e., 3 months), delivered as part of usual care, developed and offered by SporMed and managed by a multiprofessional team. The programme began and ended with an evaluation visit aimed at defining and assessing the patient's goals in collaboration with adapted PA specialists. The intervention was solely based on PA and did not include methods specifically aimed at improving the psychological variables considered (e.g., cognitive-behavioural therapy). However, some behaviour change techniques (BCTs) were used by adapted PA specialists to promote a sustainable PA practice, such as goal setting behaviour, feedback on outcomes of behaviour, social support (unspecified), instruction on how to perform a behaviour, information about health and emotional consequences, behavioural practice/rehearsal and social reward [39]. The programme consisted of 3–4 1-h group-based sessions per week. Each session included 30 min of aerobic cardiovascular exercise training at near-lipoxmax intensity (60–70% Heart Rate Reserve), on ergocycles (e.g., bicycle, handcycle), treadmills and elliptical trainers, supervised by an adapted PA specialist. The remaining 30 min were dedicated to segmental muscle strengthening of the lower and upper limbs (e.g., 3 sets of 15–20 repetitions at 50% of Maximum Volontary Contraction on 6 machines) supervised by a sports physiotherapist.

#### 2.1.4 Statistical analysis

We estimated the sample size a priori based on similar studies to ensure comparability. Following Lakens' recommendations [40], we used the smallest effect size from Megakli et al. (BODY effect size, Cohen's *f* = 0.20) for power calculations. To detect this effect size with 90% power, a sample size of 46 was needed, accounting for four measurement points and a correlation of 0.5, with an alpha of 0.05. To account for a potential 10% dropout rate, we aimed to include 51 participants. Given a dropout rate exceeding 10% during data collection (see Fig. 1), we increased the sample size to improve our chances of detecting an effect.

Statistical analyses were performed using R (R Foundation for Statistical Computing, Vienna, Austria). Attrition analyses were conducted to compare completers and non-completers at baseline on self-perception variables, motivational profiles according to self-determination theory, and BMI. A correlation analysis was conducted as the primary analysis (see Table 1). Multilevel models were computed for each of the outcomes (i.e., GSE, PSW, COND, SPORT, STREN, BODY, body dissatisfaction, body appreciation) to analyse within-person evolution across time. Initially, we tested a random intercepts multilevel model without predictors. This null model served as a comparative baseline for evaluating the predictive improvement of the models that include predictors (i.e., BMI, age, time). The independent time variable was coded either continuously in some models (see Table S1) or categorically to observe potential changes between time measurements. Since the measurement times during the programme were one month apart, while three months separated the end of the programme and the follow-up, these two sequences were treated independently (T0–T3 and T3–T4). Changes in psychological outcomes were evaluated using models including time as a categorical variable and confounding factors (i.e., age, BMI) as fixed effects. BMI and age were tested as predictors of self-perceptions variables. Predictor variables were grand-mean centered. Random intercepts for participants and random linear slopes for repeated measures at the participant level were considered. When significant changes over time were detected, we conducted contrast analyses and computed effect sizes (Cohen's *d*) with 95% confidence intervals.

**Table 1:** Means, standard deviations, and Pearson's correlations between the Study 1 variables of the sample (n = 63) at T0 measurement.

Variable	*M*	*SD*	1	2	3	4	5	6	7	8	9
1. GSE	2.24	0.77									
2. PSW	2.27	1.01	**0.41∗∗**								
3. COND	1.59	0.71	0.15	**0.50∗∗**							
4. SPORT	2.02	1.00	0.24	**0.46∗∗**	**0.34∗∗**						
5. BODY	2.07	0.96	**0.72∗∗**	**0.42∗∗**	0.24	**0.27∗**					
6. STREN	1.83	1.02	**.33∗∗**	**0.47∗∗**	**0.44∗∗**	**0.64∗∗**	**0.50∗∗**				
7. Body dissatisfaction	4.04	0.82	**-0.26∗**	−0.21	0.07	−0.06	**-0.38∗∗**	−0.09			
8. Body appreciation	2.48	0.72	**0.68∗∗**	**0.55∗∗**	**0.32∗**	**0.27∗**	**.076∗∗**	**0.48∗∗**	**-0.52∗∗**		
9. BMI	40.29	6.13	0.18	−0.11	−0.21	−0.03	0.19	0.18	−0.04	0.11	
10. Age	44.87	13.05	−0.09	−0.01	−0.07	−0.18	−0.07	−0.18	−0.11	0.01	**-0.28∗**

*Note.* GSE = Global self-esteem; PSW = Physical self-worth; COND = Physical condition; SPORT = Sports competence; BODY = Body attractiveness; STREN = Physical strength; BMI = Body Mass Index. *M* and *SD* are used to represent mean and standard deviation, respectively. ∗*p* < .05. ∗∗*p* < .01.

Given the number of outcomes and time contrasts reported in the manuscript, we conducted a multiple-testing sensitivity analysis in line with recent recommendations [41]. In a sensitivity analysis, the Benjamini–Hochberg procedure was applied to the unadjusted p-values to control the false discovery rate [42]. Corrections were conducted separately within each study and within four conceptually distinct, prespecified families of comparisons: primary baseline-to-post-intervention effects (T0–T3), exploratory adjacent month-to-month changes, post-intervention-to-follow-up changes (T3–T4), and baseline-to-follow-up effects (T0–T4).

### 2.2 Study 1 results

#### 2.2.1 Descriptive results

Among the 155 women assessed for eligibility, 102 were allocated to the intervention, but 39 did not complete it (see Fig. 1). Baseline attrition analyses showed that completers and non-completers were comparable for self-perception variables, motivational profiles according to self-determination theory, and BMI, except for COND, which differed between groups (p = .03). The final sample of completers comprised 63 women aged 19–78 years (*M*_age_ = 44.9 years, SD = 13.1) with a height between 153 and 178 cm (*M*_height_ = 164.3 cm, SD = 6.6) and a weight between 82 and 201 kg (*M*_weight_ = 109.1 kg, SD = 20.3). The BMI ranged from 31.2 to 64.9 kg/m^2^ (M_BMI_ = 40.3, SD = 6.1). Regarding obesity class distribution, most participants were classified as having class II obesity (n = 29, 46.0%), followed by class III obesity (n = 26, 41.3%) and class I obesity (n = 8, 12.7%). Previous bariatric surgery was documented in 8 participants (12.7%), whereas 55 participants (87.3%) reported no previous bariatric procedure. Women attended an average of n = 14.2 (SD = 3.9) sessions during the first month of the intervention, n = 11.5 (SD = 4.4) during the second month, and n = 9.9 (SD = 4.7) during the third and final month of the programme.

#### 2.2.2 Preliminary analysis

GSE was positively correlated with PSW and body appreciation and negatively associated with body dissatisfaction (*ps* < 0.05) (see Table 1). PSW was positively correlated with its four subdomains: COND, SPORT, BODY, and STREN (*ps* < 0.01). It was also positively associated with body appreciation (*p* < 0.01). Body dissatisfaction and body appreciation were negatively correlated (*p* < 0.01). None of the psychological constructs correlated with BMI or age.

#### 2.2.3 Main analysis

The means and standard deviations of the psychological study variables for each measurement are presented in Table 2.

**Table 2:** Unadjusted means and standard deviations of Study 1 variables for each measurement for participants in the face-to-face intervention.

		GSE M (SD)	PSW M (SD)	COND M (SD)	SPORT M (SD)	STREN M (SD)	BODY M (SD)	Body dissatisfaction M (SD)	Body appreciation M (SD)
Pre face-to-face intervention	T0	2.24 (0.77)	2.27 (1.00)	1.59 (0.71)	2.01 (1.00)	1.83 (1.02)	2.07 (0.96)	4.04 (0.82)	2.48 (0.72)

During face-to-face intervention	T1	2.29 (0.87)	3.19 (0.96)	1.86 (0.77)	2.39 (0.84)	2.02 (0.97)	2.32 (1.09)	3.66 (0.80)	2.69 (0.72)
	T2	2.43 (0.89)	3.36 (0.86)	2.05 (0.72)	2.61 (0.90)	2.35 (1.09)	2.37 (1.09)	3.67 (0.97)	2.72 (0.73)
	T3	2.56 (1.04)	3.48 (0.93)	2.17 (0.88)	2.59 (0.94)	2.23 (1.04)	2.47 (1.19)	3.54 (0.90)	2.81 (0.77)
Post face-to face intervention	T4	2.77 (1.26)	3.39 (1.21)	1.99 (0.98)	2.66 (1.06)	2.28 (1.12)	2.62 (1.36)	3.47 (1.14)	2.99 (0.90)

*Note.* GSE = Global self-esteem; PSW = Physical self-worth; COND = Physical condition; SPORT = Sports competence; BODY = Body attractiveness; STREN = Physical strength. *M* and *SD* are used to represent mean and standard deviation, respectively.

*Evolution throughout the supervised PA programme.* As shown in Table 3, multilevel models conducted for each psychological study variable indicated a significant change over time between the start and the end of the programme (T0–T3). Contrast analyses revealed a significant increase in GSE (*d* = 0.81, 95% CI [0.42, 1.20], *p* < .001), PSW (*d* = 1.90, 95% CI [1.52, 2.28], *p* < .001), each of its subdomains (COND: *d* = 1.14, 95% CI [0.76, 1.52], *p* < .001; SPORT: *d* = 1.03, 95% CI [0.66, 1.41], *p* < .001; STREN: *d* = 0.76, 95% CI [0.37, 1.14], *p* < 0.001; BODY: *d* = 1.01, 95% CI [0.62, 1.40], *p* < .001), and body appreciation (*d* = 1.12, 95% CI [0.73, 1.51], *p* < .001) whereas body dissatisfaction (*d* = −0.99, 95% CI [−1.37, −0.61], *p* < .001) significantly decreased. More precisely, the change was significant within the first month of the programme for each of the variables (T0–T1), i.e., PSW (*d* = 1.39, 95% CI [1.02, 1.76], *p* < .001), COND (*d* = 0.53, 95% CI [0.17, 0.89], *p* < .01), SPORT (*d* = 0.67, 95% CI [0.31, 1.04], *p* < .001), STREN (*d* = 0.35, 95% CI [0.01, 0.72], *p* = 0.05), BODY (*d* = 0.56, 95% CI [0.19, 0.93], *p* < .01), except for GSE (*p* = 0.67). During the second month (T1–T2), only STREN significantly improved (*d* = 0.54, 95% CI [0.16, 0.91], *p* < .01), while only GSE significantly improved (*d* = 0.41, 95% CI [0.02, 0.80], *p* < .05) in the third and final month of the programme (T2–T3).

**Table 3:** Multilevel analysis of predictors of global self-esteem, its physical domain and subdomains, and body facets: Estimates of fixed and random effects during and post-face-to-face programme.

	GSE	PSW	COND	SPORT	STREN	BODY	Body dissatisfaction	Body appreciation
	Est (SE)	Est (SE)	Est (SE)	Est (SE)	Est (SE)	Est (SE)	Est (SE)	Est (SE)
Fixed effects during the programme								

Intercept	−0.16 (0.12)	**−0.74∗∗∗** (0.11)	**−0.38∗∗∗** (0.12)	**−0.39∗∗∗** (0.11)	**−0.26∗** (0.12)	−0.23 (0.12)	**0.34∗∗** (0.12)	**−0.26∗** (0.12)
T0–T1	0.06 (0.09)	**0.87∗∗∗** (0.11)	**0.33∗∗** (0.11)	**0.40∗∗∗** (0.11)	**0.18∗** (0.09)	**0.21∗∗** (0.07)	**−0.38∗∗∗** (0.10)	**0.28∗∗∗** (0.07)
T0–T2	**0.19∗** (0.09)	**1.03∗∗∗** (0.12)	**0.55∗∗∗** (0.12)	**0.60∗∗∗** (0.11)	**0.46∗∗∗** (0.10)	**0.30∗∗∗** (0.07)	**−0.42∗∗∗** (0.11)	**0.33∗∗∗** (0.08)
T0–T3	**0.38∗∗∗** (0.09)	**1.18∗∗∗** (0.12)	**0.71∗∗∗** (0.12)	**0.61∗∗∗** (0.11)	**0.39∗∗∗** (0.10)	**0.38∗∗∗** (0.07)	**−0.56∗∗∗** (0.11)	**0.46∗∗∗** (0.08)
T1–T2	0.13 (0.09)	0.17 (0.12)	0.22 (0.12)	0.20 (0.11)	**0.28∗∗∗** (0.10)	0.09 (0.07)	−0.04 (0.11)	0.05 (0.08)
T1–T3	**0.33∗∗∗**(0.09)	**0.32∗∗∗** (0.12)	**0.38∗∗∗** (0.12)	0.22 (0.11)	**0.21∗∗∗** (0.10)	**0.17∗** (0.07)	−0.18 (0.11)	**0.18∗** (0.08)
T2–T3	**0.19∗∗∗**(0.09)	0.15 (0.12)	0.16 (0.12)	0.02 (0.11)	−0.07 (0.10)	0.08 (0.07)	−0.13 (0.11)	0.13 (0.08)
Age	−0.05 (0.11)	**−0.19∗** (0.09)	**−0.23∗∗** (0.09)	**−0.32∗∗∗** (0.09)	**−0.24∗** (0.10)	−0.10 (0.10)	−0.06 (0.11)	0.04 (0.11)
BMI	0.11 (0.11)	−0.05 (0.09)	**−0.20∗** (0.10)	−0.14 (0.10)	0.00 (0.10)	0.13 (0.11)	0.01 (0.11)	0.05 (0.11)

Random effects during the programme								

Conditional R^2^	0.77	0.61	0.60	0.63	0.72	0.85	0.69	0.82
Residual variance	0.22	0.39	0.39	0.35	0.27	0.14	0.31	0.17
Between-indiv. variance	0.71	0.36	0.44	0.44	0.59	0.78	0.65	0.77
Intra-class correlation coeff.	0.76	0.48	0.53	0.56	0.69	0.85	0.67	0.82

Fixed effects post-programme								

Intercept	−0.10 (0.13)	0.04 (0.13)	0.08 (0.12)	−0.03 (0.12)	−0.00 (0.12)	−0.09 (0.13)	0.08 (0.13)	−0.12 (0.13)
T3–T4	0.12 (0.09)	−0.13 (0.12)	**−0.30∗∗** (0.09)	0.03 (0.11)	−0.01 (0.10)	0.10 (0.07)	−0.08 (0.10)	**0.21∗∗** (0.08)
Age	−0.08 (0.13)	−0.22 (0.13)	**−0.29∗** (0.13)	**−0.47∗∗∗** (0.12)	**−0.36∗∗** (0.12)	−0.16 (0.13)	−0.00 (0.13)	−0.06 (0.13)
BMI	0.01 (0.13)	−0.12 (0.13)	**−0.28∗** (0.13)	−0.13 (0.12)	−0.01 (0.12)	0.04 (0.13)	−0.03 (0.13)	0.10 (0.13)

Random effects post-programme								

Conditional R^2^	0.82	0.69	0.82	0.77	0.80	0.88	0.79	0.88
Residual variance	0.17	0.30	0.18	0.23	0.20	0.11	0.21	0.12
Between-indiv. variance	0.75	0.63	0.69	0.57	0.65	0.80	0.77	0.80
Intra-class correlation coeff.	0.81	0.67	0.79	0.71	0.77	0.88	0.79	0.87

*Note.* GSE = Global self-esteem; PSW = Physical self-worth; COND = Physical condition; SPORT = Sports competence; BODY = Body attractiveness; STREN = Physical strength; BMI = Body Mass Index. *Est* and *SE* are used to represent Estimates and Standard Error, respectively. ∗*p* < .05; ∗∗*p* < .01; ∗∗∗*p* < .001.

On the other hand, a significant decrease in BMI was observed between T0 and T3 (*d* = −1.46, 95% CI [−1.83, −1.08], *p* < .001).

*Evolution during the follow-up.* As shown in Table 3, most of the variables investigated (i.e., GSE, PSW, SPORT, STREN, BODY, body dissatisfaction and BMI) remain unchanged between the end of the programme and the three-month follow-up (T3–T4). Nevertheless, COND significantly decreased (*d* = −0.71, 95% CI [−1.16, −0.26], *p* < .01) whereas body appreciation significantly increased (*d* = 0.62, 95% CI [0.17, 1.08], *p* < .01) at follow-up. Despite the few changes observed at follow-up, each of the variables remains significantly different from the pre-programme measures (*ps* < 0.01).

*Self-perceptions predictors.* Furthermore, age and to a lesser extent BMI appear to be predictors of the evolution of certain (sub)domains of self-esteem but not body image. Age was significantly and negatively associated with PSW, COND, SPORT, and STREN (*ps* < 0.05), both in the models including T0–T3 and T3–T4 data, except for PSW in the latter (see Table 3). In other words, older participants tended to score lower on these variables. BMI was significantly and negatively associated with COND both in the models including T0–T3 and T3–T4 data (*ps* < 0.05), suggesting a lower self-assessment in participants with a higher BMI. Finally, the interaction between time and BMI was not significant for any of the psychological variables (see Table S1), whether during or after the programme.

The Benjamini–Hochberg sensitivity analysis produced a pattern of results that was broadly consistent with the primary analyses. In Study 1, 8/8 baseline-to-post-intervention effects, 7/9 month-to-month effects (out of 24 contrasts tested), 2/2 post-intervention-to-follow-up effects (out of 8 contrasts), and 8/8 baseline-to-follow-up effects remained statistically significant after correction. The complete raw and Benjamini–Hochberg-adjusted p-values are reported in Table S3.

### 2.3 Study 1 discussion

The purpose of Study 1 was to examine the month-by-month effects of a three-month face-to-face supervised PA programme on GSE, PSW and its subdomains, body dissatisfaction, and body appreciation in a sample of women with obesity. The extent to which potential effects observed during the intervention programme persisted three months after its completion was also studied.

The main findings regarding the evolution throughout the programme revealed that participants who completed the intervention showed significant improvements. More specifically, the levels of GSE, PSW, COND, SPORT, STREN, BODY, and body appreciation increased between the beginning and the end of the programme, while body dissatisfaction decreased. Our hypothesis was supported and results are in line with those obtained by Megakli et al. [12] regarding self-esteem (except for BODY) and provide new insights into the evolution of body dissatisfaction and body appreciation.

However, an examination of the month-by-month evolution of each psychological variable during the programme reveals that, after significant changes in the first month, most variables plateaued for the rest of the programme (except for GSE and STREN), which differed from the findings in Megakli et al.’s study (2017).

During the three months of follow-up, body appreciation continued to increase significantly. This result is promising given the challenge of achieving lasting effects in obesity management [43] and warrants further investigation to understand why it is the only variable to improve after the programme. Moreover, each variable was maintained at a higher level at follow-up compared to the pre-programme level, which confirms the results of Megakli et al. [12]. Our hypothesis was indeed partially supported. This maintenance may be attributed to the fact that most participants (i.e., 81.8%) reported continuing to engage in PA after the programme, either with supervision or independently.

## 3 Study 2

### 3.1 Study 2 methods

Study 2 was a prospective longitudinal observational cohort study evaluating changes during participation in an existing digital supervised physical activity programme.

#### 3.1.1 Participants and procedure

**Fig. 2:**
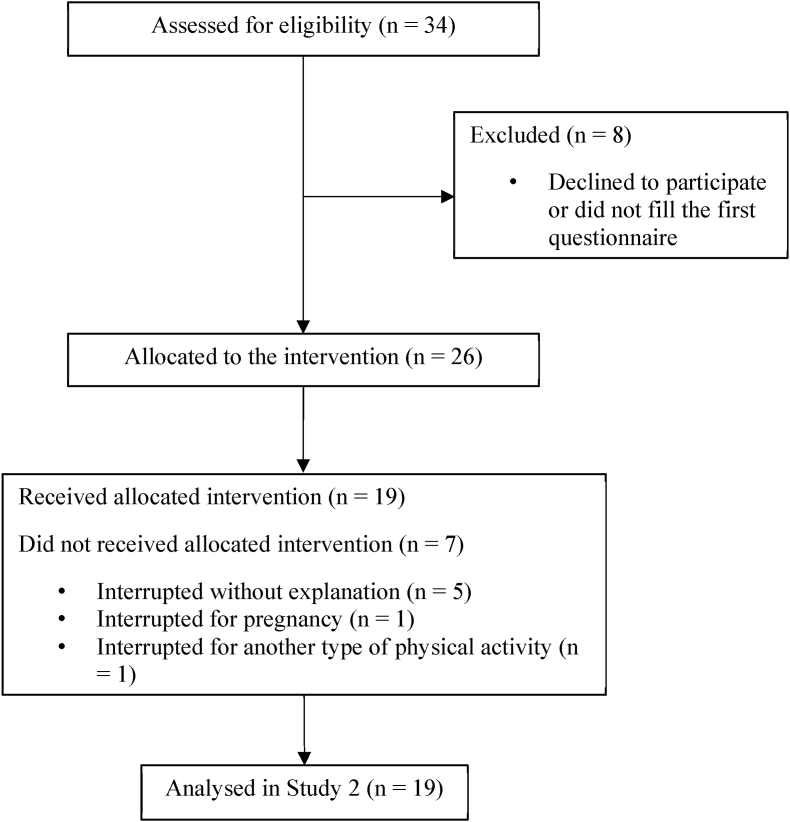
Participants flowchart for the digital intervention.

The inclusion and non-inclusion criteria, as well as the procedure, including informed consent, were the same as in Study 1. An additional inclusion criterion was to have access to a smartphone or computer with an Internet connection to independently use the KIPLIN® app. As in Study 1, participants who did not complete the entire three-month programme (dropout rate = 26.31 %; see Fig. 2) were not included in the final analyses.

To ensure transparency, the TIDieR reporting guidelines were followed. Study 2 was approved by Rennes 2 ethics Committee (n°2019-019). Data were collected using a longitudinal design between March 2023 and February 2024.

#### 3.1.2 Measures

All survey instruments were the same as in Study 1.

*Demographic, anthropometric variables*. Information on age, and self-reported weight and height were collected enabling BMI to be calculated.

*Global self-esteem and physical self-perceptions*. In Study 2, reliability was acceptable for most subscale: α = 0.84, 0.78, 0.77, and 0.87 for PSW, SPORT, STREN, and BODY components respectively, but questionable for the GSE (α = 0.60) and COND (α = 0.61) subscales. These limitations are further addressed in the discussion section.

*Body dissatisfaction*. In Study 2, α = 0.91 for BSQ-8C.

*Body appreciation*. In Study 2, α = 0.94 for BAS-2.

*Adherence*. Participants were asked to report the number of sessions attended in the past month at T1, T2, and T3, and to indicate whether they had taken part in the monthly gamified activity.

#### 3.1.3 Description of the PA intervention

Participants were enrolled in the existing KIPLIN® intervention as part of usual care, a group-based digital programme that relies on gamification strategies. Available as an app on smartphones, tablets, or computers, the 12-week (i.e., 3 months) intervention comprised four components: (1) a remote supervised PA programme with telecoaching sessions led by adapted PA specialists, (2) a gamification of PA via multiple games (3) an activity monitoring tool (i.e., a pedometer), and (4) a chat interface for discussion. For each participant, the intervention began and ended with a remote evaluation session conducted by an adapted PA specialist. The live remote supervised PA programme consisted of at least 1 1-h telecoaching session per week with the option to register for additional weekly meetings. Each session was limited to seven participants, who registered based on their preferences and availability. The programme included aerobic, cardiovascular, muscle-strengthening, and stretching exercises, and periodically featured playful elements (e.g., quizzes) to provide information on the benefits of PA. Additionally, participants completed one 14-day PA game each month, in which daily step counts were converted to points used to progress through the game. They had access to this self-monitoring tool via the app as a percentage of objective – progressively increasing – achieved. Finally, the chat interface aimed to encourage peers to interact with each other, creating social interactions. All these features are part of the standard KIPLIN® app and are described in greater detail in the protocol study of another trial, along with the 16 behaviour change theories and techniques (BCTs) implemented (e.g., social support, 3.1 – team challenges where participants must collaborate to progress in the game [44]). Although no objective PA measures were collected in the present study, evidence from a prior RCT conducted in the same population has demonstrated the KIPLIN® intervention's effectiveness in increasing daily steps and moderate-to-vigorous PA relative to a supervised face-to-face adapted PA programme [24]. Consistent with these findings, real-world data from a large retrospective analysis of KIPLIN® users showed that the intervention was associated with increased daily steps, particularly among individuals with lower baseline activity levels [45].

#### 3.1.4 Statistical analysis

Given the exploratory nature of the study, the sample size was determined by recruitment capacity during the inclusion period rather than by a priori power calculations. To assess the risk of a Type II error for the effects of interest, we conducted a sensitivity power analysis. Drawing on studies in relative comparable populations and designs [12,32,46], we estimated the minimum detectable effect size for the present sample of n = 19, assuming five measurement points, a correlation of 0.5, and an alpha level of 0.05 with 80% power. Results indicated that the present sample was adequately powered to detect medium effects (Cohen's *f* ≥ 0.25 approximately) for the following variables: GSE, PSW, COND, SPORT, STREN, BODY, body appreciation, and body dissatisfaction. Smaller effects, if present, would likely remain undetected. Readers should therefore interpret non-significant findings in Study 2 with this constraint in mind, as they may reflect insufficient statistical power rather than a genuine absence of effect.

The data analytic strategy was the same as Study 1. The independent time variable was coded either continuously in some models (see Table S2) or categorically to observe potential changes between time measurements.

### 3.2 Study 2 results

#### 3.2.1 Descriptive results

Among the 34 women assessed for eligibility, 26 were allocated to the intervention and 7 did not complete it. Baseline attrition analyses showed that completers and non-completers did not differ significantly in self-perception variables or BMI. Differences were observed for motivational regulations, with non-completers reporting higher levels of external regulation (p = .02) and amotivation (p = .01) compared with completers. The final sample of completers comprised 19 women aged 23–61 years (*M*_age_ = 41.4 years, SD = 11.2) with a height range of 153–178 cm (*M*_height_ = 165.6 cm, SD = 6.0) and a weight between 82 and 170 kg (*M*_weight_ = 112.6 kg, SD = 20.8). BMI ranged from 30.8 to 58.8 kg/m^2^ (M_BMI_ = 41.1, SD = 7.4). Regarding obesity class distribution, most participants were classified as having class II obesity (n = 9, 47.4%), followed by class III obesity (n = 8, 42.1%) and class I obesity (n = 2, 10.5%). Among the participants, 8 (42.1%) had previously undergone bariatric surgery, whereas 11 (57.9%) had not undergone any bariatric procedure. Participants attended an average of 3.84 sessions (SD = 2.74) during the first month, 2.31 (SD = 2.10) during the second, and 2.63 (SD = 3.35) during the third. Over the same three periods, the percentages of participants who completed the 14-day PA game were 73.7%, 57.9%, and 47.4%, respectively.

#### 3.2.2 Main analysis

The means and standard deviations of the psychological study variables for each measurement are presented in Table 4.

**Table 4:** Unadjusted means and standard deviations of Study 2 variables for each measurement for participants in the digital intervention.

		GSE M (SD)	PSW M (SD)	COND M (SD)	SPORT M (SD)	STREN M (SD)	BODY M (SD)	Body dissatisfaction M (SD)	Body appreciation M (SD)
Pre digital intervention	T0	2.39 (0.86)	2.42 (1.02)	1.39 (0.57)	2.05 (0.83)	1.79 (1.02)	2.45 (1.14)	3.68 (1.18)	2.81 (0.98)

During digital intervention	T1	2.56 (0.98)	2.97 (1.09)	1.64 (0.68)	2.28 (0.94)	2.00 (0.89)	2.86 (1.35)	3.42 (1.20)	2.99 (0.85)
	T2	2.78 (0.88)	3.19 (1.02)	1.69 (0.67)	2.28 (0.86)	2.00 (0.91)	2.97 (1.32)	3.32 (1.23)	2.96 (0.97)
	T3	2.65 (0.90)	2.84 (1.04)	1.68 (0.77)	2.18 (0.84)	2.03 (0.92)	2.97 (1.23)	3.16 (1.24)	3.06 (0.90)
Post digital intervention	T4	2.86 (1.04)	3.27 (1.49)	1.60 (0.69)	2.33 (1.01)	2.07 (1.15)	2.93 (1.27)	2.94 (1.03)	3.26 (0.88)

*Note.* GSE = Global self-esteem; PSW = Physical self-worth; COND = Physical condition; SPORT = Sport competence; BODY = Body attractiveness; STREN = Physical strength. *M* and *SD* are used to represent mean and standard deviation, respective.

*Evolution throughout the digital PA intervention.* As shown in Table 5, multilevel analyses indicated that most variables increased over time between the start and the end of the intervention (T0–T3). Contrast analyses revealed an increase in PSW (*d* = 0.80, 95% CI [0.10, 1.50], *p* < .05), COND (*d* = 0.88, 95% CI [0.19, 1.57], *p* < .05), BODY (*d* = 1.24, 95% CI [0.53, 1.95], *p* < .01) and body appreciation (*d* = 0.83, 95% CI [0.13, 1.54], *p* < .05), and a decrease in body dissatisfaction (*d* = −1.08, 95% CI [−1.78, −0.39], *p* < .01).

**Table 5:** Multilevel analysis of predictors of global self-esteem, its physical domain and subdomains, and body facets: Estimates of fixed and random effects during and post-digital programme.

	GSE	PSW	COND	SPORT	STREN	BODY	Body dissatisfaction	Body appreciation
	Est (SE)	Est (SE)	Est (SE)	Est (SE)	Est (SE)	Est (SE)	Est (SE)	Est (SE)
Fixed effects during the programme								

Intercept	−0.23 (0.22)	−0.41 (0.22)	−0.30 (0.22)	−0.17 (0.23)	−0.20 (0.22)	−0.30 (0.22)	0.24 (0.22)	−0.17 (0.22)
T0–T1	0.13 (0.17)	**0.48∗∗** (0.17)	**0.32∗** (0.15)	0.23 (0.15)	0.20 (0.18)	**0.27∗** (0.11)	−0.19 (0.13)	0.15 (0.12)
T0–T2	**0.44∗** (0.17)	**0.70∗∗∗** (0.17)	**0.38∗** (0.15)	0.22 (0.16)	0.25 (0.18)	**0.44∗∗∗** (0.12)	**−0.29∗** (0.14)	0.18 (0.12)
T0–T3	0.31 (0.17)	**0.42∗** (0.17)	**0.40∗∗** (0.15)	0.14 (0.15)	0.30 (0.18)	**0.43∗∗∗** (0.11)	**−0.44∗∗** (0.13)	**0.29∗** (0.12)
T1–T2	0.30 (0.17)	0.22 (0.18)	0.06 (0.15)	−0.01 (0.16)	0.05 (0.18)	0.16 (0.12)	−0.09 (0.14)	0.04 (0.12)
T1–T3	0.18 (0.17)	−0.06 (0.18)	0.08 (0.15)	−0.09 (0.14)	0.10 (0.18)	0.16 (0.12)	−0.25 (0.14)	0.15 (0.12)
T2–T3	−0.13 (0.17)	−0.28 (0.17)	0.02 (0.15)	−0.08 (0.15)	0.05 (0.18)	−0.01 (0.11)	−0.15 (0.13)	0.11 (0.12)
Age	−0.07 (0.20)	0.01 (0.19)	−0.05 (0.20)	−0.11 (0.21)	−0.08 (0.20)	−0.11 (0.21)	0.08 (0.20)	−0.10 (0.21)
BMI	0.15 (0.19)	0.19 (0.19)	−0.25 (0.19)	−0.11 (0.20)	0.17 (0.19)	0.09 (0.19)	−0.11 (0.19)	0.19 (0.19)

Random effects during the programme								

Conditional R^2^	0.73	0.72	0.79	0.78	0.71	0.88	0.82	0.87
Residual variance	0.21	0.31	0.09	0.16	0.21	0.19	0.24	0.10
Between-indiv. variance	0.53	0.69	0.31	0.55	0.47	1.27	1.06	0.65
Intra-class correlation coeff.	0.71	0.69	0.77	0.77	0.69	0.87	0.82	0.86

Fixed effects post-programme								

Intercept	−0.10 (0.23)	−0.14 (0.22)	0.07 (0.21)	−0.06 (0.22)	−0.02 (0.22)	0.02 (0.23)	0.09 (0.23)	−0.12 (0.22)
T3–T4	0.27 (0.15)	0.22 (0.20)	−0.28 (0.19)	0.11 (0.17)	0.01 (0.17)	0.02 (0.17)	−0.16 (0.17)	0.31 (0.15)
Age	−0.13 (0.22)	0.03 (0.21)	−0.01 (0.20)	0.03 (0.21)	−0.05 (0.21)	−0.08 (0.23)	0.05 (0.22)	−0.08 (0.22)
BMI	−0.01 (0.20)	−0.12 (0.20)	−0.26 (0.19)	−0.14 (0.19)	−0.00 (0.20)	−0.15 (0.21)	−0.07 (0.20)	0.11 (0.20)

Random effects post-programme								

Conditional R^2^	0.82	0.68	0.71	0.77	0.76	0.79	0.82	0.87
Residual variance	0.16	0.48	0.14	0.17	0.23	0.34	0.24	0.10
Between-indiv. variance	0.71	0.96	0.30	0.55	0.70	1.21	1.06	0.65
Intra-class correlation coeff.	0.81	0.66	0.68	0.76	0.76	0.78	0.82	0.86

*Note.* GSE = Global self-esteem; PSW = Physical self-worth; COND = Physical condition; SPORT = Sport competence; BODY = Body attractiveness; STREN = Physical strength; BMI = Body Mass Index. *Est* and *SE* mean Estimates and Standard Error, respectively. Estimates are unstandardized. ∗*p* < .05; ∗∗*p* < .01; ∗∗∗*p* < .001.

More precisely, the change occurred within the first month (T0–T1) for PSW (*d* = 0.92, 95% CI [0.22, 1.62], *p* < .05), COND (*d* = 0.71, 95% CI [0.01, 1.41], *p* < .05), and BODY (*d* = 0.79, 95% CI [0.08, 1.50], *p* < .05). None of the psychological variables studied changed, neither during the second (T1–T2) nor during the third and final month of the intervention (T2–T3).

On the other hand, a significant decrease in BMI was observed between T0 and T3 (*d* = −1.12, 95% CI [−1.82, −0.42], *p* < .01).

*Evolution during the follow-up.* As shown in Table 5, no variables – included BMI – changed during the follow-up (T3–T4); however, contrasts analyses showed that initial improvements remained significant 3-month post-intervention (T0–T4) for GSE (*d* = 0.99, 95% CI [0.25, 1.74], *p* < .05), PSW (*d* = 1.20, 95% CI [0.45, 1.95], *p* < .01), BODY (*d* = 1.07, 95% CI [0.31, 1.84], *p* < .01), body appreciation (*d* = 1.50, 95% CI [0.72, 2.27], *p* < .001), body dissatisfaction (*d* = −1.61, 95% CI [−2.37, −0.85], *p* < .001), and BMI (*d* = −1.20, 95% CI [−1.95, −0.45], *p* < .01).

*Self-perceptions predictors.* Neither age nor BMI emerged as significant predictors of the investigated psychological variables. Furthermore, the interaction between time and BMI was not significant for any of the psychological variables (see Table S2), whether during – except for STREN – or after the intervention.

The Benjamini–Hochberg sensitivity analysis produced a pattern of results that was broadly consistent with the primary analyses, except for T0-T1 significant changes. In Study 2, 5/5 baseline-to-post-intervention effects (out of 8 contrasts tested), 0/3 month-to-month effects (out of 24 contrasts tested), 0/0 post-intervention-to-follow-up effects (out of 8 contrasts), and 5/5 baseline-to-follow-up effects remained statistically significant after correction. The complete raw and Benjamini–Hochberg-adjusted p-values are reported in Table S4.

### 3.3 Study 2 discussion

Given the exploratory nature and limited sample size of Study 2, the observed effect sizes should be interpreted cautiously, and the results should be considered preliminary and hypothesis-generating. Although the sensitivity analysis using a Benjamini–Hochberg correction supported the overall pattern of the main findings, some month-to-month changes, particularly those observed between T0 and T1, were no longer statistically significant after adjustment. These temporal patterns should therefore be interpreted with caution.

Most outcomes showed medium-to-large improvements from baseline to post digital intervention, with several effects maintained at 3-month follow-up (notably PSW, BODY, body appreciation, and reduced body dissatisfaction). Given the magnitude of the effects, these changes may be clinically meaningful. Nevertheless, wide confidence intervals reflect the small sample and warrant cautious interpretation. In addition, specific month-to-month patterns analysis indicated that, after gains in the first month, PSW, COND, and BODY plateaued for the remainder of the digital intervention, suggesting that most change may occur early in the programme. This result may be explained by high adherence to both supervised PA sessions and gamified components, which then declined over time, although this interpretation remains speculative and requires further investigation. From a psychological standpoint, offering shorter, more frequent booster digital programmes could help optimize these variables in participants. In addition, this hypothesis is consistent with evidence suggesting that shorter gamified interventions may be more effective in promoting behaviour change [45]. However, it should be examined in adequately powered confirmatory studies. Our findings are broadly consistent with previous evidence supporting the role of PA interventions in improving self-esteem and body image among women with obesity [12,32]. However, subdomain analyses revealed divergent patterns: BODY scores increased significantly, while STREN and SPORT remained stable over the intervention.

At 3-month follow-up, each of the psychological variables that showed improvement during the intervention – except for COND – was maintained at a higher level compared to the pre-intervention level. These insights confirm the results of Megakli et al. [12], who reported maintenance for most variables, and a decrease in COND, one month post-programme. The maintenance may be attributed to continued PA participation post-intervention, reported by 80% of participants.

## 4 General discussion

The two studies reported in this paper, which focused on participants who completed the respective programmes, were not designed to be compared. Differences in sample size, session frequency, and intervention context should therefore be considered when interpreting the results across studies. That said, the parallel structure of both studies reveals a broadly consistent pattern: supervised PA, whether delivered face-to-face or digitally, was associated with improvements in self-esteem and body image in women with obesity, with most observed gains emerging within the first month of the programme. The Benjamini–Hochberg sensitivity analysis did not materially alter the overall pattern of findings, supporting the robustness of the principal intervention effects. However, some month-to-month changes observed in Study 2 no longer reached statistical significance after correction, suggesting that these specific temporal patterns should be interpreted cautiously. The present study did not assess the mechanisms underlying these psychological changes. Therefore, the following interpretations should be considered as hypotheses that may guide future research. On the other hand, where divergences exist, notably the absence of significant SPORT and STREN improvements in Study 2, these differences may reflect intervention-specific constraints.

Supervised PA is known to induce changes in body composition such as reduction in body fat and BMI [19]. These changes, whether related to PA practice or to other factors, are often associated with improved self-perceptions in individuals with obesity [12,47]. However, and interestingly, our results suggest that changes in self-perceptions were not determined by weight loss in the participants studied. Several potential mechanisms may be hypothesized to explain the positive effects observed in each intervention. Firstly, it has been demonstrated that supervised PA increases muscle strength and cardiorespiratory capacity in individuals with obesity [48]. If participants experienced those improvements, they may have contributed to the observed changes in physical self-esteem and body image facets. However, in Study 2, neither STREN nor SPORT showed significant changes. This discrepancy with Study 1 and with existing literature [12] may stem from the low frequency of sessions actually completed by participants as well as the inherent limitations imposed by the telecoaching sessions, which limit access to strength-training equipment and opportunities to develop sport-specific skills.

Each supervised PA programme was tailored to participants’ abilities and skills through the establishment of achievable goals, thereby fostering successful experiences, notably through the gamification features embedded within the KIPLIN® intervention. One possible explanation for the improvement in self-esteem variables during each intervention is that it may be related to increased exercise self-efficacy, which positively influences physical subdomains according to the Exercise and Self-Esteem Model [49]. Such effects have been previously documented in both face-to-face [12] and digital settings [50] among women with obesity, although the digital intervention in the latter study incorporated a multimodal approach. It is plausible that a bottom-up process occurred, whereby improvements in physical subdomains and – particularly – BODY [49], preceded an increase in PSW, followed by GSE, more stable over time [4]. Moreover, another hypothesis is that perceived rather than actual physical changes – which better predict improvements in body image [51] – may have contributed to the observed reduction in body dissatisfaction across all two intervention modalities. Self-esteem and body image are both social constructs influenced by interpersonal experiences [7,52]. Therefore, one possible explanation is that forming groups comprised solely of individuals with obesity who share the same stigmatizing characteristic may have facilitated the development of peer relationships and a feeling of membership. According to the social identity approach [53], the emergence of shared identities within each group-based programme may have enhanced a greater sense of belonging, social connectivity, and social cohesion. This, in turn, could have helped participants overcome stereotype-related barriers [44,54], and view themselves, including their body, more positively.

One of the most consistent and theoretically interesting findings across both studies is the pattern of early, rapid improvement followed by a plateau: most psychological variables improved significantly within the first month but showed little further change thereafter. This finding diverges from Megakli et al. [12], who reported non-linear but continued gains throughout a comparable programme, and warrants careful consideration. Several hypotheses may explain this pattern and should be tested in future research. First, from a goal-setting perspective, the establishment of individually tailored and achievable targets during the evaluation session may have fostered early engagement and positive psychological outcomes, as suggested by Goal-Setting Theory [55]. However, as participants progressed through the programme, the absence of regular reassessment and adjustment of these goals– except via gamification – may have reduced opportunities for continued goal attainment and associated psychological favourable changes [56], including further improvement in self-esteem and body image facets. If so, regularly reassessing and adjusting goals throughout the programme could represent a key area for improvement, irrespective of the intervention modality. Second, the early weeks of a structured group programme typically involve heightened novelty, social novelty, and increased practitioner attention toward new participants. This concentrated social and motivational input may produce an initial boost in self-perceptions that is difficult to sustain as participants and practitioners settle into routine. The aforementioned social identity processes (i.e., the emergence of shared identity and group cohesion) may similarly consolidate quickly and then stabilise rather than continuing to grow linearly. Third, the decline in session attendance observed across months in both studies may have reduced the frequency of competence-affirming experiences necessary to drive further gains. If the initial improvements could be partly driven by the novelty and intensity of early engagement, reduced adherence over time would predictably attenuate further progress.

These findings suggest the potential value of three-month interventions for improving self-perceptions in individuals with obesity. Importantly, the results of both studies highlight that the first month of a PA programme may represent a critical window of psychological plasticity for women with obesity. Intervention designers might consider sequencing and prioritizing important interventional features and BCTs such as goal setting, social support, or performance accomplishment from the onset of the intervention [57] to optimize these effects. In addition, since the progression of most variables who had evolved seems to settle down after the first few weeks, it is worth considering how to strengthen this dynamic during the second and third months of the intervention to increase its effectiveness, regardless of the delivery modality. Moreover, as the present study was not designed to compare intervention modalities, future randomized controlled trials including both face-to-face and remote delivery conditions would be needed to determine whether these changes are comparable across formats.

## 5 Limitations

Given the limited sample size in Study 2, the lack of detailed demographic information, and the reliance on self-reported measures, the present findings should be interpreted with caution. The dropout rate might have an impact on the results presented in the study, although it is quite common in weight management interventions for adults with obesity [58]. Attrition analyses comparing completers and non-completers on baseline self-perception and motivational profiles (according to self-determination theory), as well as BMI, indicated that the two groups were largely comparable with the exception of COND in the face-to-face programme. In the digital programme, differences were only observed for motivational regulations, with non-completers reporting higher levels of external regulation and amotivation than completers. These findings suggest that baseline differences between completers and non-completers were limited, although residual attrition bias cannot be ruled out. Several threats to internal validity remain unaddressed by our within-group longitudinal repeated-measures design. Specifically, we cannot rule out regression to the mean — whereby participants with the most impaired baseline self-perceptions may have improved regardless of intervention — nor can we exclude the possibility that heightened attention and structured social contact inherent in group-based programmes (i.e., a Hawthorne-type effect) contributed to the observed improvements independently of the physical activity content. Objective longitudinal measures of physical activity, cardiorespiratory fitness, muscular strength, body composition, and functional capacity were not collected. In addition, longitudinal BMI estimates were primarily based on self-reported weight. This limits our ability to determine whether the observed psychological changes were accompanied by objectively measured changes in physical activity, fitness, strength, or body composition. Future research should build on these findings by including larger, more diverse samples to confirm these (exploratory) analyses, investigate factors limiting changes beyond the first month of intervention and optimize the psychological favourable changes associated with PA participation.

## 6 Conclusion

The two independent studies reported here provide preliminary and complementary evidence that completing a supervised physical activity intervention — whether delivered face-to-face or digitally — was associated with meaningful improvements in self-esteem, body appreciation and body dissatisfaction among women with obesity. Specifically, participants who completed either programme showed significant gains in most self-esteem variables, body appreciation, and reductions in body dissatisfaction, with these improvements largely maintained at three-month follow-up. Although Study 2 provides initial evidence that similar favourable changes may also occur in a digital setting, these findings are hypothesis-generating and require replication in larger, adequately powered studies. The present work also provides a more detailed month-by-month description of changes in self-perception over the course of the programmes. We observed in both studies the concentration of psychological gains within the first month of each programme, followed by a plateau for the remainder of the intervention. This pattern suggests that the opening weeks of a supervised PA programme may represent a critical moment for self-esteem, body appreciation and body dissatisfaction. It also highlights the need for further investigation into the underlying mechanisms to maximize the psychological favourable changes of such intervention.

• Supervised physical activity programmes, whether delivered face-to-face or digitally, can produce clinically meaningful improvements in self-esteem, body appreciation, and body dissatisfaction among women with obesity, with changes largely maintained after programme completion.
• The first month of intervention appears to be a critical therapeutic window, as most psychological improvements occur early in the programme, highlighting the importance of maximizing engagement and support during this initial phase.
• Given the established links between self-perception, mental health, long-term physical activity adherence, and weight management, integrating supervised physical activity into obesity care may provide favourable psychological and behavioural changes that support sustained health outcomes.

These findings appear particularly relevant given the rising obesity epidemic, as self-perception variables are recognized as key indicators of mental health and predictors of sustained PA engagement and long-term weight loss in patients.

## CRediT author statement

LM, DLF, AM, and GC contributed to the study according to the CRediT taxonomy, including conceptualization, methodology, investigation, data curation, formal analysis, and writing (original draft and review & editing). LM, DLF, and GC contributed to the study design. All authors contributed to data acquisition, analysis and interpretation, and approved the final version of the manuscript.

## Research data

The datasets supporting the findings of this study are publicly available on Zenodo at the following DOI: https://doi.org/10.5281/zenodo.20345390.

## Ethics review

These studies were approved by the Rennes 2 Ethics Committee (Protocol No. 2019-019). They evaluated existing programmes delivered as routine clinical care and were not registered as prospective clinical trials. All participants received information regarding study procedures, risks, benefits, confidentiality, and their right to withdraw at any time without consequence. Electronic informed consent was obtained prior to participation. Study 1 was preregistered at https://aspredicted.org/N5D_KG2.

## Declaration of artificial intelligence (AI) and AI-assisted technologies

During the preparation of this work the author used AI to translate sentences. After using this tool/service, the author reviewed and edited the content as needed and takes full responsibility for the content of the publication.

## Funding

With the support of the ANR within the framework of the PIA EUR DIGISPORT Project (ANR-18-EURE-0022).

## Declaration of competing interests

The authors declare that they have no competing interests.
