# Supplementary material for: Changes in self-esteem, body dissatisfaction, and body appreciation during face-to-face and digital supervised physical activity interventions in women with obesity: A dual-study investigation
Source: Obes Pillars. 2026 Jul 16;19:100303. doi: 10.1016/j.obpill.2026.100303 (PMC13396761; doi:10.1016/j.obpill.2026.100303)
Supplement: Multimedia component 1 [file mmc1.docx]

***Supplementary materials***

Table S1. Multilevel analysis of continuously coded predictors of global self-esteem, its physical domain and subdomains: Estimates of fixed and random effects during and post face-to-face programme.

|  | GSE | PSW | COND | SPORT | STREN | BODY | Body dissatisfaction | Body appreciation |
| --- | --- | --- | --- | --- | --- | --- | --- | --- |
|  | Est (SE) | Est (SE) | Est (SE) | Est (SE) | Est (SE) | Est (SE) | Est (SE) | Est (SE) |
| Fixed effects during the programme |  |  |  |  |  |  |  |  |
| Intercept | -0.01 (0.11) | 0.01 (0.09) | 0.00 (0.09) | 0.01 (0.09) | -0.01 (0.10) | -0.02 (0.11) | 0.01 (0.11) | -0.00 (0.11) |
| Time | **0.14***** (0.03) | **0.42***** (0.04) | **0.27***** (0.04) | **0.23***** (0.04) | **0.16***** (0.04) | **0.14***** (0.03) | **-0.19***** (0.04) | **0.16***** (0.03) |
| Age | -0.05 (0.11) | **-0.20*** (0.09) | **-0.24*** (0.09) | **-0.33***** (0.09) | **-0.25**** (0.10) | -0.10 (0.10) | -0.05 (0.11) | 0.04 (0.11) |
| BMI | 0.12 (0.11) | -0.07 (0.09) | **-0.21*** (0.10) | -0.15 (0.10) | -0.00 (0.10) | 0.13 (0.11) | 0.02 (0.11) | 0.04 (0.11) |
| Time x BMI | -0.01 (0.03) | 0.07 (0.04) | 0.06 (0.04) | 0.06 (0.04) | 0.00 (0.03) | -0.01 (0.03) | -0.04 (0.04) | 0.02 (0.03) |
| Random effects during the programme |  |  |  |  |  |  |  |  |
| Conditional R² | 0.77 | 0.57 | 0.61 | 0.62 | 0.71 | 0.85 | 0.69 | 0.82 |
| Residual variance | 0.22 | 0.43 | 0.39 | 0.36 | 0.27 | 0.14 | 0.32 | 0.17 |
| Between-indiv. variance | 0.70 | 0.35 | 0.44 | 0.44 | 0.60 | 0.78 | 0.65 | 0.77 |
| Intra-class correlation coeff. | 0.76 | 0.45 | 0.53 | 0.55 | 0.68 | 0.84 | 0.67 | 0.82 |
| Fixed effects post-programme |  |  |  |  |  |  |  |  |
| Intercept | -0.05 (0.12) | -0.01 (0.12) | -0.05 (0.12) | -0.02 (0.11) | -0.01 (0.12) | -0.05 (0.12) | 0.04 (0.13) | -0.03 (0.12) |
| Time | 0.06 (0.05) | -0.06 (0.06) | **-0.15**** (0.05) | 0.02 (0.05) | -0.00 (0.05) | 0.05 (0.04) | -0.04 (0.05) | **0.10**** (0.04) |
| Age | -0.08 (0.13) | -0.21 (0.13) | **-0.29*** (0.13) | **-0.46***** (0.12) | **-0.36**** (0.12) | -0.16 (0.13) | -0.01 (0.13) | -0.06 (0.13) |
| BMI | 0.02 (0.13) | -0.12 (0.13) | **-0.27*** (0.13) | -0.12 (0.12) | -0.02 (0.12) | 0.04 (0.13) | -0.04 (0.13) | 0.09 (0.13) |
| Time x BMI | -0.06 (0.04) | -0.05 (0.06) | -0.04 (0.04) | -0.07 (0.05) | 0.01 (0.05) | -0.02 (0.04) | 0.03 (0.05) | 0.04 (0.04) |
| Random effects post-programme |  |  |  |  |  |  |  |  |
| Conditional R² | 0.82 | 0.70 | 0.83 | 0.78 | 0.80 | 0.88 | 0.79 | 0.88 |
| Residual variance | 0.16 | 0.30 | 0.18 | 0.22 | 0.20 | 0.11 | 0.20 | 0.11 |
| Between-indiv. variance | 0.75 | 0.63 | 0.70 | 0.57 | 0.65 | 0.80 | 0.76 | 0.81 |
| Intra-class correlation coeff. | 0.82 | 0.68 | 0.80 | 0.72 | 0.77 | 0.88 | 0.79 | 0.88 |

*Note.* GSE = Global self-esteem; PSW = Physical self-worth; COND = Physical condition; SPORT = Sport competence; BODY = Body attractiveness; STREN = Physical strength; BMI = Body Mass Index. *Est* and *SE* are used to represent Estimates and Standard Error, respectively. ^*^ *p* < .05; ^**^ *p* < .01; ^***^p < .001.

Table S2. Multilevel analysis of continuously coded predictors of global self-esteem, its physical domain and subdomains: Estimates of fixed and random effects during and post face-to-face programme.

|  | GSE | PSW | COND | SPORT | STREN | BODY | Body dissatisfaction | Body appreciation |
| --- | --- | --- | --- | --- | --- | --- | --- | --- |
|  | Est (SE) | Est (SE) | Est (SE) | Est (SE) | Est (SE) | Est (SE) | Est (SE) | Est (SE) |
| Fixed effects during the programme |  |  |  |  |  |  |  |  |
| Intercept | -0.01 (0.20) | -0.02 (0.19) | -0.03 (0.20) | -0.03 (0.21) | -0.02 (0.20) | -0.01 (0.21) | 0.01 (0.21) | -0.01 (0.21) |
| Time | **0.18*** (0.07) | **0.29***** (0.07) | **0.17**** (0.06) | 0.09 (0.06) | 0.10 (0.06) | **0.18***** (0.05) | **-0.12*** (0.05) | 0.08 (0.05) |
| Age | -0.06 (0.20) | 0.00 (0.19) | -0.04 (0.20) | -0.13 (0.21) | -0.05 (0.20) | -0.11 (0.21) | 0.11 (0.21) | -0.11 (0.21) |
| BMI | 0.15 (0.20) | 0.26 (0.19) | -0.18 (0.20) | -0.04 (0.20) | 0.24 (0.20) | 0.12 (0.20) | -0.12 (0.20) | 0.21 (0.20) |
| Time x BMI | -0.04 (0.07) | -0.01 (0.07) | -0.09 (0.06) | -0.09 (0.06) | **-0.15*** (0.07) | 0.04 (0.05) | 0.03 (0.05) | 0.01 (0.05) |
| Random effects during the programme |  |  |  |  |  |  |  |  |
| Conditional R² | 0.71 | 0.74 | 0.78 | 0.80 | 0.78 | 0.89 | 0.86 | 0.88 |
| Residual variance | 0.28 | 0.26 | 0.21 | 0.19 | 0.22 | 0.11 | 0.13 | 0.12 |
| Between-indiv. variance | 0.63 | 0.58 | 0.69 | 0.75 | 0.68 | 0.81 | 0.75 | 0.79 |
| Intra-class correlation coeff. | 0.69 | 0.69 | 0.77 | 0.80 | 0.75 | 0.88 | 0.85 | 0.87 |
| Fixed effects post-programme |  |  |  |  |  |  |  |  |
| Intercept | 0.01 (0.22) | -0.04 (0.20) | -0.05 (0.20) | -0.01 (0.21) | -0.02 (0.21) | 0.01 (0.21) | 0.04 (0.21) | 0.00 (0.21) |
| Time | 0.13 (0.08) | 0.11 (0.10) | -0.14 (0.09) | 0.05 (0.08) | 0.00 (0.09) | 0.00 (0.08) | -0.07 (0.08) | 0.15 (0.07) |
| Age | -0.13 (0.22) | 0.03 (0.21) | 0.00 (0.20) | 0.04 (0.21) | -0.06 (0.21) | -0.12 (0.22) | 0.08 (0.22) | -0.11 (0.21) |
| BMI | -0.01 (0.20) | -0.12 (0.20) | -0.24 (0.19) | -0.14 (0.19) | -0.01 (0.20) | -0.18 (0.20) | -0.05 (0.20) | 0.09 (0.19) |
| Time x BMI | -0.02 (0.08) | -0.01 (-0.01) | 0.07 (0.10) | 0.01 (0.09) | -0.02 (0.09) | -0.19 (0.09) | 0.12 (0.09) | -0.14 (0.08) |
| Random effects post-programme |  |  |  |  |  |  |  |  |
| Conditional R² | 0.82 | 0.68 | 0.72 | 0.77 | 0.76 | 0.81 | 0.80 | 0.84 |
| Residual variance | 0.18 | 0.31 | 0.26 | 0.21 | 0.22 | 0.19 | 0.20 | 0.15 |
| Between-indiv. variance | 0.77 | 0.61 | 0.59 | 0.68 | 0.68 | 0.76 | 0.74 | 0.75 |
| Intra-class correlation coeff. | 0.81 | 0.66 | 0.69 | 0.76 | 0.75 | 0.80 | 0.79 | 0.83 |

Table S3. Benjamini-Hochberg sensitivity analysis results for Study 1.

| study | family | outcome | contrast | p raw | p BH | retained BH |
| --- | --- | --- | --- | --- | --- | --- |
| Study 1 | primary | GSE | T0 - T3 | **0.0000** | **0.0001** | **true** |
| Study 1 | primary | PSW | T0 - T3 | **0.0000** | **0.0000** | **true** |
| Study 1 | primary | COND | T0 - T3 | **0.0000** | **0.0000** | **true** |
| Study 1 | primary | SPORT | T0 - T3 | **0.0000** | **0.0000** | **true** |
| Study 1 | primary | BODY | T0 - T3 | **0.0000** | **0.0000** | **true** |
| Study 1 | primary | STREN | T0 - T3 | **0.0001** | **0.0001** | **true** |
| Study 1 | primary | Body dissatisfaction | T0 - T3 | **0.0000** | **0.0000** | **true** |
| Study 1 | primary | Body appreciation | T0 - T3 | **0.0000** | **0.0000** | **true** |
| Study 1 | monthly | GSE | T0 - T1 | 0.5168 | 0.5672 | false |
| Study 1 | monthly | GSE | T1 - T2 | 0.1428 | 0.2608 | false |
| Study 1 | monthly | GSE | T2 - T3 | 0.0366 | 0.1097 | false |
| Study 1 | monthly | PSW | T0 - T1 | **0.0000** | **0.0000** | **true** |
| Study 1 | monthly | PSW | T1 - T2 | 0.1521 | 0.2608 | false |
| Study 1 | monthly | PSW | T2 - T3 | 0.2196 | 0.2957 | false |
| Study 1 | monthly | COND | T0 - T1 | **0.0042** | **0.0166** | **true** |
| Study 1 | monthly | COND | T1 - T2 | 0.0669 | 0.1606 | false |
| Study 1 | monthly | COND | T2 - T3 | 0.1778 | 0.2845 | false |
| Study 1 | monthly | SPORT | T0 - T1 | **0.0003** | **0.0018** | **true** |
| Study 1 | monthly | SPORT | T1 - T2 | 0.0813 | 0.1774 | false |
| Study 1 | monthly | SPORT | T2 - T3 | 0.8769 | 0.8769 | false |
| Study 1 | monthly | BODY | T0 - T1 | **0.0028** | **0.0135** | **true** |
| Study 1 | monthly | BODY | T1 - T2 | 0.2013 | 0.2957 | false |
| Study 1 | monthly | BODY | T2 - T3 | 0.2841 | 0.3589 | false |
| Study 1 | monthly | STREN | T0 - T1 | 0.0542 | 0.1445 | false |
| Study 1 | monthly | STREN | T1 - T2 | **0.0052** | **0.0178** | **true** |
| Study 1 | monthly | STREN | T2 - T3 | 0.4874 | 0.5672 | false |
| Study 1 | monthly | Body dissatisfaction | T0 - T1 | **0.0003** | **0.0018** | **true** |
| Study 1 | monthly | Body dissatisfaction | T1 - T2 | 0.6896 | 0.7196 | false |
| Study 1 | monthly | Body dissatisfaction | T2 - T3 | 0.2218 | 0.2957 | false |
| Study 1 | monthly | Body appreciation | T0 - T1 | **0.0002** | **0.0018** | **true** |
| Study 1 | monthly | Body appreciation | T1 - T2 | 0.5200 | 0.5672 | false |
| Study 1 | monthly | Body appreciation | T2 - T3 | 0.1196 | 0.2392 | false |
| Study 1 | follow-up | GSE | T3 - T4 | 0.2214 | 0.4429 | false |
| Study 1 | follow-up | PSW | T3 - T4 | 0.2880 | 0.4607 | false |
| Study 1 | follow-up | SPORT | T3 - T4 | 0.7535 | 0.8611 | false |
| Study 1 | follow-up | COND | T3 - T4 | **0.0030** | **0.0243** | **true** |
| Study 1 | follow-up | STREN | T3 - T4 | 0.9386 | 0.9386 | false |
| Study 1 | follow-up | BODY | T3 - T4 | 0.2089 | 0.4429 | false |
| Study 1 | follow-up | Body dissatisfaction | T3 - T4 | 0.4293 | 0.5724 | false |
| Study 1 | follow-up | Body appreciation | T3 - T4 | **0.0094** | **0.0376** | **true** |
| Study 1 | baseline to follow-up | GSE | T0 - T4 | **0.0001** | **0.0001** | **true** |
| Study 1 | baseline to follow-up | PSW | T0 - T4 | **0.0000** | **0.0000** | **true** |
| Study 1 | baseline to follow-up | COND | T0 - T4 | **0.0036** | **0.0036** | **true** |
| Study 1 | baseline to follow-up | SPORT | T0 - T4 | **0.0000** | **0.0000** | **true** |
| Study 1 | baseline to follow-up | BODY | T0 - T4 | **0.0000** | **0.0000** | **true** |
| Study 1 | baseline to follow-up | STREN | T0 - T4 | **0.0001** | **0.0001** | **true** |
| Study 1 | baseline to follow-up | Body dissatisfaction | T0 - T4 | **0.0000** | **0.0000** | **true** |
| Study 1 | baseline to follow-up | Body appreciation | T0 - T4 | **0.0000** | **0.0000** | **true** |

Table S4. Benjamini-Hochberg sensitivity analysis results for Study 2.

| study | family | outcome | contrast | p raw | p BH | retained BH |
| --- | --- | --- | --- | --- | --- | --- |
| Study 2 | primary | GSE | T0 - T3 | 0.0781 | 0.1042 | false |
| Study 2 | primary | PSW | T0 - T3 | **0.0209** | **0.0335** | **true** |
| Study 2 | primary | COND | T0 - T3 | **0.0117** | **0.0313** | **true** |
| Study 2 | primary | SPORT | T0 - T3 | 0.3792 | 0.3792 | false |
| Study 2 | primary | BODY | T0 - T3 | **0.0006** | **0.0047** | **true** |
| Study 2 | primary | STREN | T0 - T3 | 0.1060 | 0.1211 | false |
| Study 2 | primary | Body dissatisfaction | T0 - T3 | **0.0023** | **0.0092** | **true** |
| Study 2 | primary | Body appreciation | T0 - T3 | **0.0176** | **0.0335** | **true** |
| Study 2 | monthly | GSE | T0 - T1 | 0.4439 | 0.7572 | false |
| Study 2 | monthly | GSE | T1 - T2 | 0.0960 | 0.5478 | false |
| Study 2 | monthly | GSE | T2 - T3 | 0.4732 | 0.7572 | false |
| Study 2 | monthly | PSW | T0 - T1 | 0.0092 | 0.2207 | false |
| Study 2 | monthly | PSW | T1 - T2 | 0.2283 | 0.5478 | false |
| Study 2 | monthly | PSW | T2 - T3 | 0.1183 | 0.5478 | false |
| Study 2 | monthly | COND | T0 - T1 | 0.0415 | 0.3322 | false |
| Study 2 | monthly | COND | T1 - T2 | 0.6991 | 0.9269 | false |
| Study 2 | monthly | COND | T2 - T3 | 0.9172 | 0.9538 | false |
| Study 2 | monthly | SPORT | T0 - T1 | 0.1611 | 0.5478 | false |
| Study 2 | monthly | SPORT | T1 - T2 | 0.9538 | 0.9538 | false |
| Study 2 | monthly | SPORT | T2 - T3 | 0.6352 | 0.8968 | false |
| Study 2 | monthly | BODY | T0 - T1 | 0.0236 | 0.2835 | false |
| Study 2 | monthly | BODY | T1 - T2 | 0.1856 | 0.5478 | false |
| Study 2 | monthly | BODY | T2 - T3 | 0.9532 | 0.9538 | false |
| Study 2 | monthly | STREN | T0 - T1 | 0.2764 | 0.5549 | false |
| Study 2 | monthly | STREN | T1 - T2 | 0.8110 | 0.9269 | false |
| Study 2 | monthly | STREN | T2 - T3 | 0.7780 | 0.9269 | false |
| Study 2 | monthly | Body dissatisfaction | T0 - T1 | 0.1669 | 0.5478 | false |
| Study 2 | monthly | Body dissatisfaction | T1 - T2 | 0.5102 | 0.7652 | false |
| Study 2 | monthly | Body dissatisfaction | T2 - T3 | 0.2775 | 0.5549 | false |
| Study 2 | monthly | Body appreciation | T0 - T1 | 0.2258 | 0.5478 | false |
| Study 2 | monthly | Body appreciation | T1 - T2 | 0.7750 | 0.9269 | false |
| Study 2 | monthly | Body appreciation | T2 - T3 | 0.3574 | 0.6598 | false |
| Study 2 | follow-up | GSE | T3 - T4 | 0.1132 | 0.4434 | false |
| Study 2 | follow-up | PSW | T3 - T4 | 0.3030 | 0.6060 | false |
| Study 2 | follow-up | SPORT | T3 - T4 | 0.5476 | 0.7301 | false |
| Study 2 | follow-up | COND | T3 - T4 | 0.1663 | 0.4434 | false |
| Study 2 | follow-up | STREN | T3 - T4 | 0.9508 | 0.9508 | false |
| Study 2 | follow-up | BODY | T3 - T4 | 0.9038 | 0.9508 | false |
| Study 2 | follow-up | Body dissatisfaction | T3 - T4 | 0.3838 | 0.6141 | false |
| Study 2 | follow-up | Body appreciation | T3 - T4 | 0.0692 | 0.4434 | False |
| Study 2 | baseline to follow-up | GSE | T0 - T4 | **0.0087** | **0.0140** | **true** |
| Study 2 | baseline to follow-up | PSW | T0 - T4 | **0.0017** | **0.0046** | **true** |
| Study 2 | baseline to follow-up | COND | T0 - T4 | 0.3880 | 0.3880 | false |
| Study 2 | baseline to follow-up | SPORT | T0 - T4 | 0.1087 | 0.1450 | false |
| Study 2 | baseline to follow-up | BODY | T0 - T4 | **0.0052** | **0.0104** | **true** |
| Study 2 | baseline to follow-up | STREN | T0 - T4 | 0.1330 | 0.1520 | false |
| Study 2 | baseline to follow-up | Body dissatisfaction | T0 - T4 | **0.0000** | **0.0004** | **true** |
| Study 2 | baseline to follow-up | Body appreciation | T0 - T4 | **0.0001** | **0.0006** | **true** |
